# One-stage simultaneous hip-preserving surgeries for the management of bilateral femoral head osteonecrosis: a mean 7.0-year follow-up

**DOI:** 10.1186/s13018-019-1467-4

**Published:** 2019-12-21

**Authors:** Wenjun Feng, Pengcheng Ye, Shihao Ni, Peng Deng, Lu Lu, Jinlun Chen, Jianchun Zeng, Xinyu Qi, Jie Li, Ke Jie, Houran Cao, Zhijun Yue, Haitao Zhang, Yirong Zeng

**Affiliations:** 1 The First Affiliated Hospital of Guangzhou University of Chinese Medicine, Jichang Road 16#, District Baiyun, Guangzhou, Guangdong China; 2 Guangzhou University of Chinese Medicine, Jichang Road 12#, District Baiyun, Guangzhou, Guangdong China; 3 The First Affiliated Hospital of Guangzhou University of Chinese Medicine, Linnan Medical Research Center of Guangzhou University of Chinese Medicine, Jichang Road 16#, District Baiyun, Guangzhou, Guangdong China; 4 Guangzhou University of Chinese Medicine, Jichang Road 16#, District Baiyun, Guangzhou, Guangdong China

**Keywords:** Osteonecrosis of the femoral head, Hip-preserving, Fibular impaction allografting, Vascularized greater trochanter bone flap, Comparative study

## Abstract

**Background:**

A retrospective study was conducted to evaluate and compare the clinical and radiological outcomes of one-stage fibular impaction allografting and vascularized greater trochanter flap autografting for the treatment of bilateral osteonecrosis of the femoral head (ONFH).

**Methods:**

Patients who underwent one-stage aforementioned hip-preserving surgeries due to bilateral ONFH were retrospectively reviewed from January 2008 to December 2013. Sixty-nine patients (138 hips) with a mean age of 31.5 years and mean follow-up of 7.0 years were included. Hips that underwent fibular impaction allografting and vascularized greater trochanter flap autografting were assigned as group A and group B, respectively. Harris Hip Score (HHS) and Visual Analogue Scale (VAS) were used for clinical evaluation, and a series of X-ray images were used for radiological assessment. For inter-group analysis, the paired *t* test was used for continuous data, and the Wilcoxon rank sum test was used for non-parametric data, while the Mann-Whitney *U* test was used for intra-group analysis.

**Results:**

The HHS and VAS in both groups A and B had a substantial advancement when compared with the preoperative level (*p* < 0.01). Fibular impaction allografting can achieve more pain relief (*p* < 0.01), though no clinical difference was found in terms of minimal clinically important difference (MCID < 10 points). Group A showed better radiological results than group B (*p* = 0.04). It was discovered that the appropriate indication for each procedure was patients with Association for Research on Osseous Circulation (ARCO) stages II and III, respectively.

**Conclusion:**

One-stage hip-preserving surgeries for the management of bilateral ONFH could obtain good medium and long-term outcomes. It was recommended that fibular impaction allografting is more suitable for patients in ARCO stage II, while for patients in ARCO stage III, vascularized greater trochanter flap autografting is a better preference.

**Trial registration:**

Retrospectively registered.

## Introduction

Osteonecrosis of the femoral head (ONFH) mainly affects young individuals in their thirties and forties [1]. Although multiple risk factors have been identified [2], such as alcohol intake, corticosteroid abuse, and trauma, the underlying pathophysiology has still not been well-recognized, which makes its treatment very challenging. The terminal course of ONFH is femoral head collapse, articular cartilage degeneration, and subsequent late-stage osteoarthritis when no timely treatment initiated [3, 4]. Total hip arthroplasty (THA) can resolve the condition with definite success and long-term satisfactory outcomes [5]. However, regardless of improvements in joint prosthesis design, instruments, and surgical techniques, THA is unlikely to be endured for the rest of the life of the acceptors, especially for young individuals. Thus, it is of great significance to preserve the necrotic femoral head, especially for young patients.

A lot of efforts have been made from conservative methods to surgical interventions for the management of ONFH. For those with early-stage asymptomatic femoral head necrosis, nonsurgical treatments might be effective, such as administration of antithrombotic drugs, bisphosphonates, lipid-lowering agents, and some herbals [6–9]. Non-weight-bearing of the affected hip with the assistance of crutches and physiotherapy was also reported to be useful [10]. Hip-preserving surgeries should be always in our priority when indexed indications are met. Various hip-preserving surgeries have also been investigated for those symptomatic large lesion pre-osteoarthritis cases, such as non-vascularized [11] or vascularized bone grafting [12], fibular impaction grafting [13], core decompression [14], rotational osteotomy [15], and stem cells implantation [16]. In brief, the ultimate goals for the treatment of ONFH are to maintain hip function, no pain, and delay or avoid THA conversion.

Previous studies have described that ONFH often occurred in bilateral hips and the incidence was as high as 78% [17, 18]. And it is commonly seen that those two hips are in different stages. For bilateral ONFH, Marcus et al. [19] reported that one-sided Phemister-type bone grafting for patients with Association for Research on Osseous Circulation (ARCO) I and II stages and concurrent THA for ARCO IV stage patients can achieve 90% success. Lih-Yuann et al. [20] who performed one-stage fibular grafting and THA for 36 patients concluded that patients obtain more benefits because of cost-effective and less perioperative morbidity and that the head-preserved hips had better survivorship. Zeng et al. [13] reported similar findings in 18 patients with both affected hips, in which one side underwent THA and the other hip accepted fibular grafting at one-stage. However, to our knowledge, one-stage hip-preserving surgery for both hips has been rarely reported in currently published articles. Cao et al. [21] who conducted a randomized clinical trial to compare free vascularized fibular grafting with core decompression in bilateral ONFH management found that both techniques can obtain good mid-term outcomes and the survivorship was 90.5% and 95.2%, respectively. However, there was no statistical significance between those two techniques. Besides, the enrolled cases were mostly pre-collapse asymptomatic cases, and mean follow-up was only 2 years. Thus, we conducted a retrospective study to evaluate and compare the clinical and radiological outcomes of one-stage fibular impaction allografting and vascularized greater trochanter flap autografting for the treatment of bilateral ONFH. Neither of those two procedures is a new technique. However, to our knowledge, no prior studies have been published. We asked the following questions: (1) What is the effect of one-stage fibular impaction allografting and vascularized bone flap autografting for the treatment of bilateral ONFH? (2) Is one procedure superior to the other one from the improvement of the Harris Hip Score (HHS) and Visual Analogue Scale (VAS)? (3) What is the appropriate indication for each procedure?

## Materials and methods

It was a monocentric and retrospective cohort study. Patients who underwent one-stage fibular impaction allografting and vascularized greater trochanter flap autografting due to bilateral ONFH from January 2008 to December 2013 were included. All surgeries were performed by one single experienced surgeon. The participants were all in ARCO stages II and III. All patients were informed that the data might be used for further study, and written consent was obtained preoperatively. This study was approved by the Ethics and Academy Committee of the First Affiliated Hospital of Guangzhou University of Traditional Chinese Medicine (Grant number [2017]124), and it complied with the Declaration of Helsinki. Patients diagnosed with bilateral ONFH at the age between 18 years and 45 years were included. The excluded criteria were previous surgical intervention of the affected hip, femoral neck and head fracture, hip infections, tumor, musculoskeletal disorders, hematologic diseases, unable to understand and follow postoperative instructions, and mental health deficiency.

We recruited 69 patients (138 hips) who underwent one-stage abovementioned hip-preserving surgeries for bilateral ONFH management. The fibular impaction allografting hips were assigned into group A, while the vascularized bone flap autografting hips were allocated to group B. Patients were contacted through regular revisit in the out-patient department to complete postoperative follow-up. And no patient was lost follow-up. The latest follow-up time was in May 2018, and the endpoint of hip-preserving surgeries was determined by the time of THA conversion. Data collection consisted of age, gender, height, weight, body mass index (BMI), etiology, follow-up time, operation time, blood loss, risk factors, and baseline ARCO stages. The details of the demographics were presented in Table 1 and Table 2.

**Table 1:** Baseline demographic characteristics of the enrolled participants

Characteristics	69 participants (138 hips)
Age (years; mean ± SD [range])	31.5 ± 7.5 (21–45)
Gender (male/female)	30/39
Height (cm; mean ± SD [range])	165.4 ± 6.9 (155–178)
Weight (kg; mean ± SD [range])	62.0 ± 9.2 (58–80)
BMI (mean ± SD [range])	22.7 ± 2.2 (18.8–26.0)
Follow-up (years; mean ± SD [range])	7.0 ± 1.0 (5–10)
Risk factors	
Idiopathic	14
Corticosteroid	30
Alcohol	25

**Table 2:** Baseline ARCO stage, operation time, and blood loss in groups A and B

Parameters	Group A	Group B	*p* value*
ARCO stage			0.809
IIA	12	3	
IIB	28	5	
IIC	19	5	
IIIA	4	18	
IIIB	4	18	
IIIC	2	20	
Operation time (minutes; mean ± SD [range])	50.5 ± 6.5 (41–60)	170.6 ± 7.8 (161–180)	< 0.01
Blood loss (ml; mean ± SD [range])	45.8 ± 7.5 (36–55)	180.7 ± 12.5 (165–200)	< 0.01

*ARCO*, Association for Research on Osseous Circulation. *p values indicated inter-group differences comparison. Continuous data was analyzed via paired *t* test, while non-parametric data was analyzed via the Wilcoxon rank sum test

### Surgical procedures

The procedures of fibular impaction allografting and vascularized greater trochanter flap autografting both had been described in previous studies [12, 13]. In brief, for hips in group A, a 5-cm to 6-cm incision originated from the tip of the greater trochanter to the femur distally was used. A 2.5-mm Kirschner pin was inserted directed toward the necrotic area until 0.5 cm below the subchondral bone under X-ray fluoroscopy. A customized hollow reamer was used to broach a tunnel via the positioning Kirschner pin, and a metal alloy T-shaped hand driller with different diameters and directions was used for necrotic bone complete debridement. Commercial allogeneic cancellous bone granules with a median size of 5 mm were then tightly impacted, followed by allogeneic fibular implantation through the tunnel, while for hips in group B, a modified Smith-Peterson approach with an incision of 15 cm to 20 cm in length was used. Firstly, the greater trochanter with the lateral femoral circumflex artery transverse branch was isolated and protected in cases of vascularity damage. Secondly, a window-like approach on the collapsed cartilage surface accessed to the necrotic area was made to remove all necrotic bone and tissues, and harden boundary was penetrated with 2.5-mm Kirschner pin for the purpose of blood supply provision from the unaffected region. Thirdly, the free iliac bone flap was harvested from the outer lip of the iliac crest and filled into the cavity after trimming in a matched shape. Fourthly, the predisposed vascularized greater trochanter was implanted through a groove along the femoral neck without entrapment and stretch, and lift-up cartilage surface was fixed and reshaped in more congruent morphology; the femoral head was then reduced. Patients were not allowed for weight-bearing at the first 6 weeks postoperatively, followed by gradually partial weight-bearing depending on the necrotic bone repair process, and total weight-bearing was permitted only after 6 months postoperatively. Tibial tuberosity skeletal traction was performed in the vascularized bone flap autografting hips immediately after surgery and lasted for 6 weeks, and then suffered limb skin traction was subsequently continued until 6 months postoperatively, while for the fibular impaction allografting hips, the skin traction sustained during the whole recovery period.

### Outcome evaluation

The clinical and radiological assessments were done by two independent researchers. Patients were examined at preoperative and postoperative 3, 6, and 12 months and 1 year annually thereafter. HHS and VAS were used for clinical outcome evaluation. Although HHS system is used for evaluating hip function, it is extremely difficult to distinguish the function disability from one hip to the contralateral hip when considering both hips were one-stage surgically treated in one single patient, because daily activities such as sitting, climbing stairs, putting on socks and shoes, using of public transportation, and walking ability are completed by both affected hips. Jasvinder et al. [22] reported that minimum clinically important difference (MCID) showed a reliable predictive ability of the HHS questionnaire, and defined a threshold of 16 to 18 points. However, the participants in our study were inter-group difference comparison for one same patient; we defined a MCID of 10 points on the 100-point HHS as Cao et al. [21] reported. X-ray images of anteroposterior and frog lateral view at preoperative and each postoperative were obtained for radiological evaluation in terms of femoral head collapse and necrotic region repair process. It was regarded as improved when femoral head morphology is stable with necrotic region partial or complete repair; stable was defined as no or subtle femoral head collapse and no increased necrotic region; aggravated was determined when progressive femoral head collapse, enlarged necrotic region, and presence of narrow joint space and osteoarthritis were seen. A survivor analysis was also performed. It was considered a clinical failure when subsequent THA conversion was indicated because of deteriorating pain, hip dysfunction, progressive femoral head collapse, and late-stage osteoarthritis. Postoperative complications were also recorded.

### Statistical analysis

For quantitative continuous data, it was expressed as means (± SD), while qualitative variables were summarized as count and percentage. For inter-group analysis, the paired *t* test was used for continuous data, and the Wilcoxon rank sum test was used for non-parametric data, while the Mann-Whitney *U* test was used for intra-group analysis. All analyses were performed using SPSS statistical software (version 18.0, IBM Corporation, USA). *p* values less than 0.05 were considered to be statistical significance.

## Results

The HHS and VAS in groups A and B improved from preoperative 70.7 ± 3.5 points (range, 64 to 76 points), 58.1 ± 5.0 points (range, 48 to 70 points), 4.8 ± 1.2 points (range, 3 to 9 points), and 8.0 ± 1.9 points (range, 4 to 10 points) to postoperative 92.4 ± 4.0 points (range, 80 to 98 points), 84.2 ± 5.8 points (range, 70 to 96 points) points, 1.1 ± 1.0 points (range, 0 to 4 points), and 2.5 ± 1.9 points (range, 0 to 6 points), respectively (Table 3). It revealed that postoperative HHS and VAS in both groups had a substantial advancement when compared with the preoperative level (*p* < 0.01). MCID (the mean HHS difference between groups A and B at the latest follow-up) was 8.2 points (95% confidential interval (CI), 2.4 to 23.2 points). It suggested that there was no clinical significance between these two groups, because MCID did not exceed a priori of 10 points thresholds. The delta VAS (the last follow-up postoperative scores minus preoperative scores) in group A was − 3.7 points (95% CI, − 4.9 to − 2.5 points), while in group B it was − 5.8 points (95% CI, − 7.6 to − 3.5 points); hips in group B suggested more pain alleviation (*p* < 0.01). The operation time in group B is much more than that in group A, which was 50.5 ± 6.6 min (range, 41 to 60 min) and 170.6 ± 7.8 min (range, 161 to 180 min), respectively (*p* < 0.01).

**Table 3:** Preoperative and the latest follow-up HHS and VAS in groups A and B

Parameters	Group A	*p* value*	Group B	*p* value*
Pre-HHS (mean ± SD)	70.7 ± 3.5	< 0.01	58.1 ± 5.0	< 0.01
Post-HHS (mean ± SD)	92.4 ± 4.0		84.2 ± 5.8	
Pre-VAS (mean ± SD)	4.8 ± 1.2	< 0.01	8.0 ± 1.9	< 0.01
Post-VAS (mean ± SD)	1.1 ± 1.0		2.5 ± 1.9	

*HHS*, Harris Hip Score; *VAS*, Visual Analogue Scale. *p values indicated intra-group difference comparison, and the Mann-Whitney *U* test was used

**Fig. 1:**
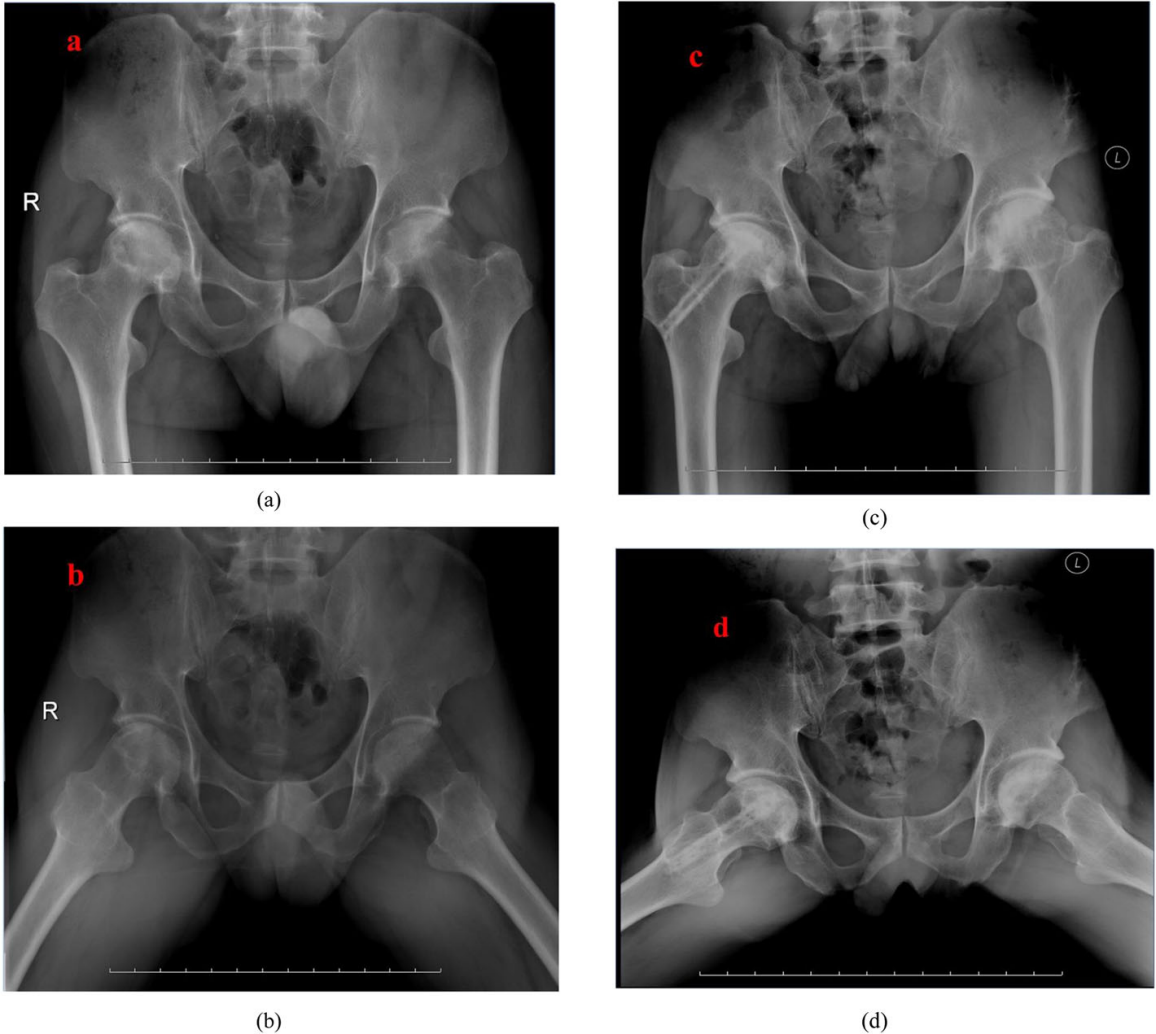
**a**, **b** 41-year-old male patient complained bilateral hip pain for 2 years. **c, d** The patient accepted one-stage fibular impaction allografting in the right hip and vascularized greater trochanter flap with the combination of cortico-cancellous iliac bone autografting. X-rays showed completed new bone formation and normal femoral head morphology without the presence of osteoarthritis at postoperative 8-year follow-up

The ARCO classification system was used for the qualitative analysis of femoral head collapse and necrotic repair process. According to abovementioned assessing criterion, 48 hips had improved results, 16 hips had stabled results, and 5 hips had aggravated results in the group A, while in the group B, there were 22 hips, 22 hips, and 25 hips in each turnover outcomes, respectively (Fig. 1). Wilcoxon test revealed that there was clinical significance between these two groups and group A showed better results (*p* = 0.04).

**Fig. 2:**
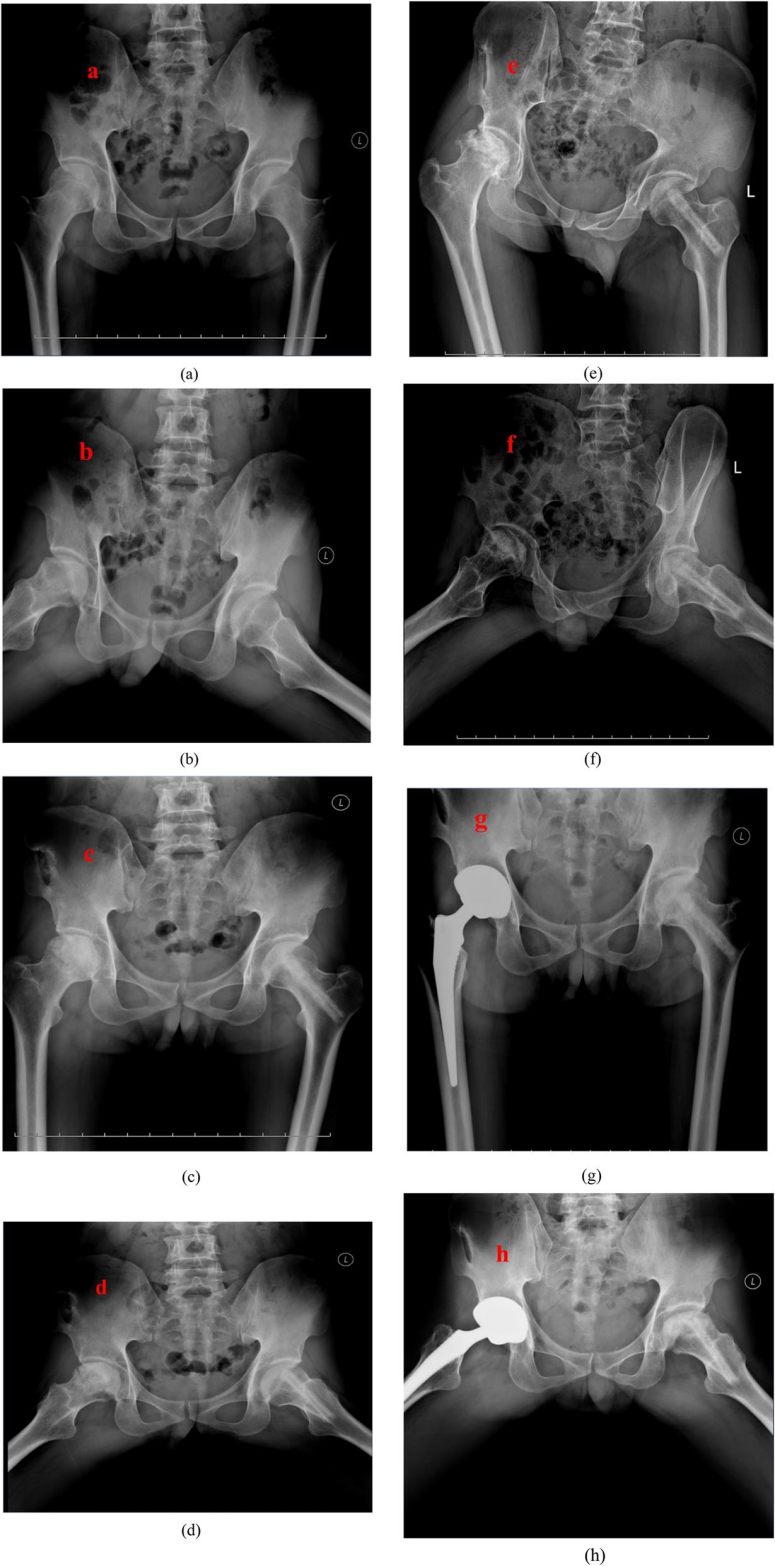
**a, b** The 38-year-old man was diagnosed with bilateral osteonecrosis of the femoral head due to alcohol overtake. **c, d** Postoperative 1-year X-rays showed both femoral head shape was maintained and the cystic area was reduced or eliminated, which indicates the necrotic repair response was good. **e, f** Postoperative 6-year X-rays revealed right femoral head collapsed and secondary osteoarthritis occurred, while the left hip was completely healed. **g, h** The patient had a right hip total hip arthroplasty conversion, while the left hip was maintained

Three hips (all were in ARCO stage IIIB) in group A need a THA conversion at a mean postoperative 5.6 years (range, 4 to 7 years). However, 10 hips received THA in group B at a mean of 6.5 years (range, 5 to 8 years) (Fig. 2). There were 1 hip in ARCO stage IIC, 3 hips in ARCO stage IIIB, and 6 hips in ARCO stage IIIC. There was 1 hip in group B that had to underwent hip-preserving revision surgery at postoperative 5 years for the improvement of hip function without pain. The patient was satisfied with the outcomes at the last 10-year follow-up. The survival rates in groups A and B were 95.7% (66/69) and 85.5% (59/69), respectively. No significance was revealed (*p* = 0.08). A slight negative correlation between the preoperative ARCO stage and the last follow-up clinical outcomes was found (Spearman correlation test, *ρ* = − 0.19, *p* = 0.04; *ρ* = − 0.28, *p* = 0.02 in groups A and B, respectively).

We further detected the appropriate indication of each procedure. The delta HHS (the latest follow-up HHS minus preoperative HHS) of groups A and B in hips with ARCO stage II (contained IIA, IIB, and IIC) and ARCO stage III (IIIA, IIIB, and IIIC) were 26.1 ± 7.0 points (95% CI, 11.9 to 36.0 points), 21.2 ± 4.7 points (95% CI, 16.8 to 28.0 points), 24.2 ± 1.1 points (95% CI, 22.5 to 25.3 points), and 27.8 ± 4.4 points (95% CI, 22.9 to 35.0 points). It showed that the appropriate indication for fibular impaction allografting was patients with ARCO stage II (*p* < 0.01), while vascularized greater trochanter flap with the combination of cortico-cancellous iliac bone autografting was the appropriate indication for patients with ARCO stage III (*p* = 0.03).

There was no complication in group A. In group B, 1 hip had superficial wound infection and finally healed when oral antibiotics were administered. Two patients complained of greater trochanter consistent mild pain, which recovered at 1 month postoperatively. No lateral femoral cutaneous nerve damage case was reported.

## Discussion

ONFH is a commonly seen disabling condition in the out-patient department, and young individuals are the main sufferers. Cui et al. [23] who conducted a multicentric epidemiologic study based on 6395 ONFH cases found that ONFH onset mostly at the age between 40 and 50 years. However, the patients enrolled in our study were mainly in their late thirties. It is difficult to discover early-stage ONFH because of concealed morbidity and asymptomatic clinical signs, and it aggravates at a rapid course. Thus, conservative treatment always seems to be not effective. Based on the symptomatic ONFH natural history, the femoral head might be progressively collapsing without operative intervention under that condition. Surgeries that can maintain the femoral head morphology, promote necrotic bone repair, and obtain good hip function, and with no pain are considered as the preferred methods for ONFH management. Many clinical trials have been reported that hip-preserving surgeries can slow down the natural history, which aims to prevent femoral head continuous collapse and avoid or delay THA conversion. However, the most-effective hip-preserving surgeries are still full of controversies and under severe debate [24, 25].

One-stage hip-preserving surgeries own the advantages of faster recovery, shorter hospital stay, and fewer costs than two-stage surgeries [26]. Regardless of the advantages, one-stage bilateral surgeries might also pose greater difficulty on the postoperative rehabilitation, and longer operation time under analgesic which may increase the risks of complications. However, we deemed that patients benefit more advantages than disadvantages from one-stage hip-preserving surgeries. We have reported that either fibular impaction allografting or vascularized greater trochanter flap autografting can obtain good results [12, 13]. Our results also showed that patients who underwent one-stage abovementioned hip-preserving surgeries can achieve great Harris Hip Score improvements and decreased VAS points. Our findings proved that one-stage hip-preserving surgeries could also be effective and safe, which could provide evidence-based complementary methods for the treatment of ONFH. The vascularity of the target vessel is the determining factor for success treatment; thus, we do not recommend traumatic patients to receive vascularized greater trochanter flap combined with autografting because the lateral femoral circumflex artery might be disrupted due to internal fixation of the displaced femoral neck fracture. However, fibular impaction allografting can achieve more pain relief, though no clinical difference was found in terms of MCID. It was probably due to the preoperative higher pain level. From the aspects of femoral head collapse and necrotic repair process, fibular impaction allografting achieved better results than combining bone grafting. It might be attributed to group A mainly having ARCO stage II hips while group B mainly having ARCO stage III hips. It is known to us that necrotic size and femoral head morphology are key factors for prognosis [27]. Besides, the survivorship at a mean 7.0-year follow-up also showed no difference. Our study suggested that the appropriate indication for fibular impaction allografting is ARCO stage II ONFH while ARCO stage III ONFH is the appropriate indication for a combination of vascularized greater trochanter with free iliac bone autografting. We found that the preoperative ARCO stage was slightly negatively correlated with the final outcomes. And alcohol and corticosteroids postoperatively sustain the status as the prognostic risk factors for THA conversion. We need more enrolled cases for further multivariate analysis. Two patients in group B complained donor site consistent mild pain, which might be attributed to the decrease of resistance to tensile stress oriented from surrounding muscles due to the greater trochanter integrity destruction.

There are numerous limitations to the study. Firstly, it was a retrospective case-cohort study. Though the evaluation was made by two independent researchers, the indexed surgeries in both hips were not randomized assigned. It might have option bias, which might affect the outcomes. Further high-level evidence randomized and double-blinded studies are required to confirm the results. Secondly, the number of included patients (69 patients with 138 hips) was relatively small. Though self-control study can minimize the impact of specific individuals, however, a large number of cases might provide better convince findings. Besides, we need a larger number of cases to perform a multivariate regression analysis to find other prognostic risk factors, which might help us to make better decisions. Thirdly, the hips in group A were mainly in ARCO II stages, while the affected hips in group B were mainly in ARCO III stages. It might be unfair to evaluate the difference between these two hip-preserving surgeries, though no baseline difference was found. Fourthly, the postoperative radiological evaluation was mainly focused on the X-ray images. Postoperative computed tomography (CT) scan and magnetic resonance image (MRI) might be essential to provide a more subjective estimation of the extent of femoral head collapse, repair process, and cartilage status.

## Conclusions

Our study concluded that one-stage fibular impaction allografting and vascularized greater trochanter flap with the combination of cortico-cancellous iliac bone autografting for the management of ONFH could obtain good medium- and long-term outcomes. And fibular impaction allografting showed better radiological results, but no clinical difference was found. We also discovered that the appropriate indication for each procedure was patients with ARCO stages II and III, respectively.

## Data Availability

All datasets used during the current study are available from the corresponding author on reasonable request.

## Abbreviations

ARCO: Association for Research on Osseous Circulation
BMI: Body mass index
HHS: Harris Hip Score
MCID: Minimal clinically important difference
ONFH: Osteonecrosis of the femoral head
THA: Total hip arthroplasty
VAS: Visual Analogue Scale
